# Acute abdominal aortic injury during posterior lumbar fusion surgery: A case report

**DOI:** 10.1097/MD.0000000000030295

**Published:** 2022-09-02

**Authors:** Jianhe Wang, Yunxiang Hu, Hong Wang

**Affiliations:** a Department of Orthopedics, Dalian Municipal Central Hospital Affiliated of Dalian Medical University, Dalian City, China; b The First Affiliated Hospital Of Dalian Medical University, Dalian City, China.

**Keywords:** case report, lumbar disc surgery, lumbar fusion surgery, vascular injury, vascular stent

## Abstract

Vascular injury is a serious complication during lumbar fusion surgery, leading to massive blood loss and life-threatening circulatory failure. In this study, we report on a patient with abdominal aorta injury at L2-L3 level during lumbar fusion surgery via posterior approach. Fortunately, our patient was successfully managed with prompt intervention.

**Patient concerns:**

A 73-year-old female was admitted to our department of low back and bilateral leg pain with claudication for over 6 months.

**Diagnosis:**

L2-S1 spinal canal stenosis, with abdominal aorta injury at the L2-L3 level during lumbar fusion surgery via a posterior approach.

**Interventions:**

L2-S1 decompression and fusion via a posterior approach was employed for spinal canal stenosis. Transluminal angioplasty with stent placement was successfully performed to stop the bleeding.

**Outcomes:**

During the procedure, it was decided that staunching the active bleeding was necessary and attention should be paid to the vital signs and blood pressure. Vascular surgical intervention was immediately scheduled when the blood pressure dropped. After stent placement, hemodynamic parameters stabilized.

**Conclusion:**

In this case report we review the prevalent sites, predisposing risk factors, diagnosis, and treatment of acute abdominal aortic injury during posterior lumbar fusion surgery, in view of our case findings. Although the incidence of vascular injury during lumbar fusion surgery is low, it is often easily overlooked. Consequently, during surgery, physicians should always be alert to the risk of vascular injury and master its clinical characteristics. Once injury is suspected, active and effective measures should promptly be taken for diagnosis and treatment to avoid serious adverse consequences.

## 1. Introduction

Lumbar fusion surgery is one of the most common surgical procedures and is frequently used to treat degenerative diseases such as lumbar disc herniation and spinal stenosis. Among these procedures, the incidence of acute vascular injury is low and could, inadvertently, be overlooked, but its mortality rate is high which usually results in serious consequences once it occurs. Harbison^1]^ concluded that the incidence of large abdominal vascular injuries complicating lumbar disc surgery is about 0.04%, but the mortality rate is as high as 15% to 61%. Complications of vascular injury during lumbar surgery, occur in most cases, at the level of L4-L5 discs.^[2–4]^ Here, we report on an abdominal aortic injury at an uncommon site, at the level of L2-L3, in a patient undergoing posterior lumbar decompression and fusion surgery. Fortunately, the injury did not cause any serious consequences due to timely detection and prompt intervention. It is tremendously worthwhile to note, that once vascular injury is suspected, active and effective measures should promptly be taken for diagnosis and treatment to avoid serious adverse consequences.

## 2. Case presentation

**Figure 1.**
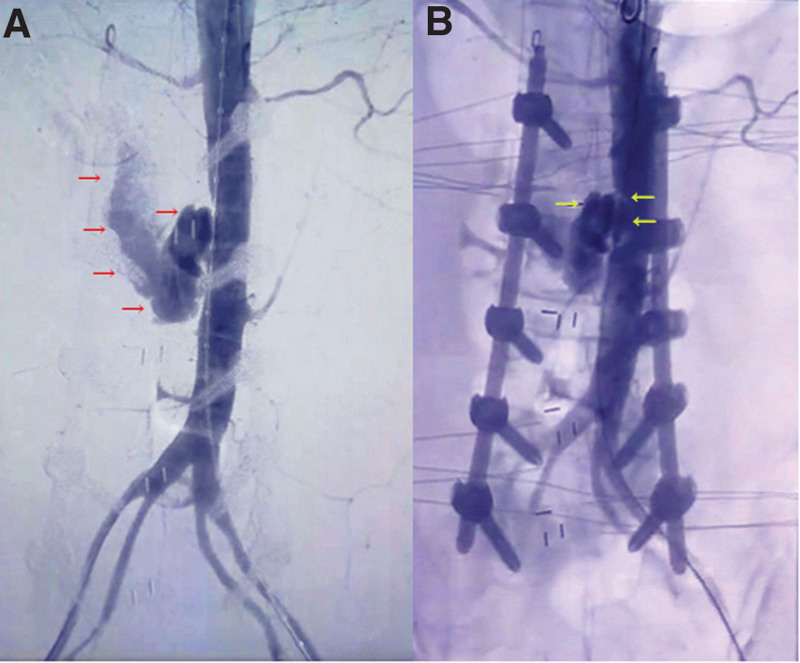
Angiography distinctly exhibits the extent of bleeding and the site of injury. (A) Red arrows point at a large amount of bleeding at the abdominal aorta. (B) Yellow arrows demonstrate bleeding from the L2-L3 intervertebral space.

**Figure 2.**
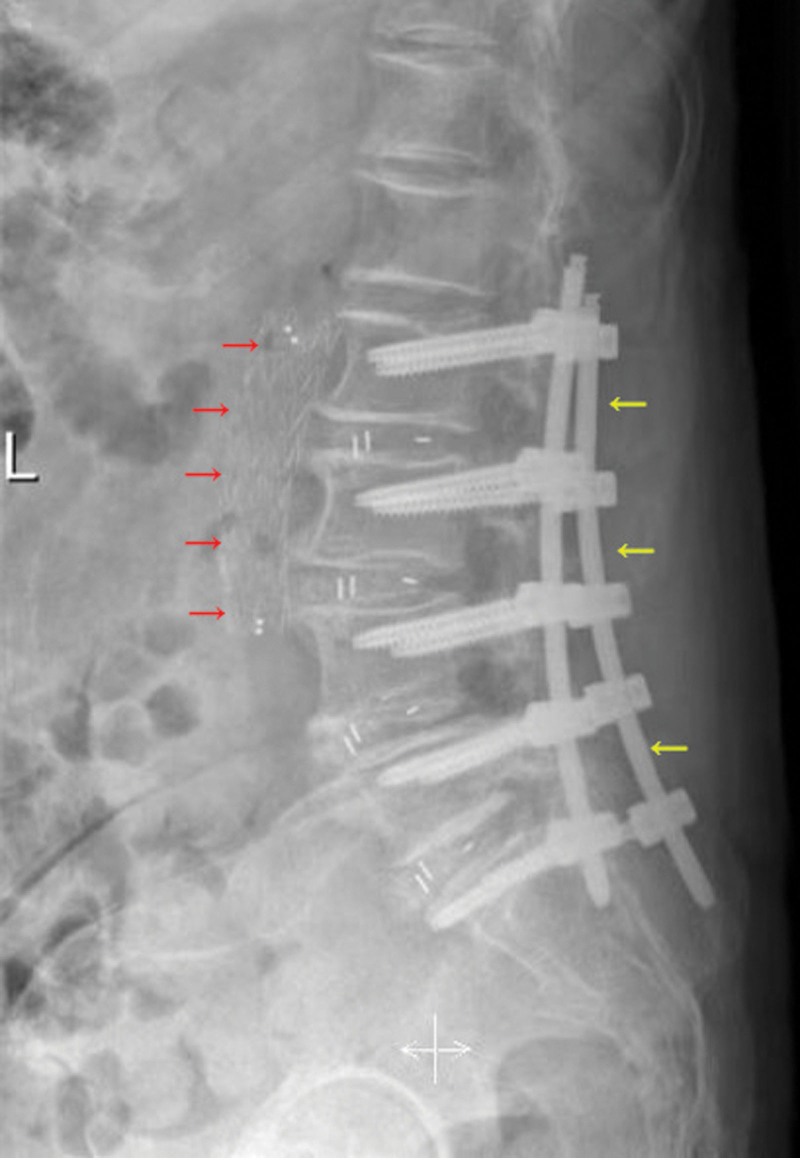
A postoperative lateral radiograph of the torso (red arrows) demonstrates a properly aligned stent inside the abdominal aorta. Yellow arrows indicate strong pedicle crew fixation from L1-S1.

A 73-year-old female was admitted to our department for low back and bilateral leg pain with claudication of over 6 months. Her body weight was 52 kg, height of 62 cm, and BMI of 19.8 kg/m^2^. No other remarkable past history was mentioned. A physical examination induced intense pain when the patient bent or twisted her spine. The. visual analogue score for both legs was 6. The patient experienced claudication within 200 m. Lower extremities demonstrated decreased muscle strength (average, level 3–4). The patient exhibited normal bowel and bladder function but had weakened bilateral knee and Achilles reflexes. Bilateral pathological reflexes were absent. Subsequently, she was diagnosed with L2-S1 spinal stenosis by computed tomography (CT) and magnetic resonance imaging and was advised to undergo L2-S1 decompression and fusion via a posterior approach. Intraoperatively, considerable bleeding was observed in the L2-L3 intervertebral space. Allogeneic transfusion was immediately given to supplement the coagulation factors and hemoglobin. Meanwhile, compression with gauze cotton was used to staunch the bleeding. Half an hour later, as no obvious active bleeding was seen in the operation field, and the blood pressure had stabilized at 100/80 mm Hg, we then completed the procedure. Postoperatively, the patient was transferred to the cardiac intensive care unit for continued rehydration and transfusion treatment. However, 2 hours after the operation, cardiac monitoring showed that the blood pressure has dropped to 60/40 mm Hg, despite massive rehydration and transfusion, with the systolic blood pressure fluctuating from 60 to 80 mm Hg. The possibility of active bleeding was again considered, so vascular surgeons were consulted, and they later performed an abdominal arteriography. Figure 1 showed the active bleeding site in the abdominal aorta. Figure 2 demonstrated transluminal angioplasty and successful stent placement. Fortunately, the patient was discharged after 2 weeks of treatment. Upon discharge, all pain symptoms were alleviated, vital signs were stable, without other signs of discomfort. The patient was discharged with instructions to dress the wound regularly, avoid exercise, and take analgesia medication when needed. The patient was followed-up on 1-week, 2-week, 1-month, and 3-month intervals. On the third month of follow-up, no obvious waist and leg discomfort were noted. Physical examination showed that the wound had healed well, lumbar mobility was normal, without tenderness nor percussion pain, and all cranial nerves were normal. No changes were noted on recent radiographs compared to those taken right after the procedure. Each vertebral body was well-fixated, and the vascular stent was stable and firm.

## 3. Discussion

The incidence of vascular injury is relatively high during posterior interbody fusion.^[5]^ Vascular complications during posterior surgery occur in 75% of cases at the L4-L5 disc, because the L4-L5 disc is the most common site of disc herniation, and the bifurcation of the bilateral iliac arteries and iliac veins are located just in front of this site.^[2]^ When the patient is in the prone position, only the anterior longitudinal ligament separates the blood vessels from the vertebral body, which is most likely to incur damage during surgery.^[6]^ Therefore, when surgical instruments break through the anterior longitudinal ligament during surgery, they can easily damage the adjacent vessels.

The main reasons for intraoperative vascular injury are as follows: the most common reason is that the surgeon does not judge the depth of the vertebral body and the vertebral space correctly, and the nucleus pulposus forceps are used with excessive depth, penetrating the anterolateral fibrous ring and the anterior longitudinal ligament and over-clamping the anterior tissue, resulting in tearing of the anterior vessels.^[7,8]^ Forceps placement should not exceed 3 cm, especially <4.5 cm. Improper patient positioning, especially when soft pillows are placed under the abdomen to alter lumbar lordosis, which reduces the space between the vessels and the disc and its ability to avoid the nucleus pulposus forceps, allowing it to be exposed to vascular injury. Additionally, sudden changes in patient position can also lead to vascular injury. Harbison^[1]^ reported a case in which a patient hiccupped intraoperatively, resulting in bleeding due to passive penetration of the anterior longitudinal ligament by the nucleus pulposus forceps. Patients with a history of previous disc surgery, abdominal surgery, etc, exhibit local fibrous tissue hyperplasia resulting in adhesions between the vessels and the vertebral body and disc. Chronic disc pathology or disc degeneration, etc, leads to increased fragility of the anterior longitudinal ligament or anterior fibrous annulus, making it easy for surgical instruments to break through the disc and anterior longitudinal ligament and thereby injure the vessels.^[8]^ Moreover, long-term vascular compression brought about by osteophytes at the anterior edge of the vertebral body and anterior disc herniation can lead to increased vascular fragility,^[9,10]^ amplifying the risk of vascular injury. Even proliferated sharp bones can directly puncture the vessel wall and form an arteriovenous fistula.^[11]^

Early diagnosis is critical for the treatment of vascular injury. When unexplained intervertebral haemorrhage suddenly occurs intraoperatively or when vascular wall tissue is found within the nucleus pulposus, it is suggestive of vascular injury.^[9,12]^ In addition, if there is an unexplained persistent drop in blood pressure or other manifestations of haemorrhagic shock, even if the amount of intervertebral bleeding is small, the possibility of vascular injury should still be highly suspected and the patient should be kept in the operation room for observation. Abdominal aspiration of noncoagulated blood is the gold standard for the diagnosis of vascular injury.^[13]^ However, it is worth noting that the elastic flap action of the intervertebral disc prevents massive blood loss and creates only a small amount of bleeding, resulting in some patients with vascular injury who do not bleed from the intervertebral space,^[14]^ and even if they do bleed, it is easy to stop the bleeding with gelatine sponges or cotton pads, which gives the illusion that no large vessels were damaged, thus delaying the diagnosis.^[15]^ Plain CT and enhanced CT are rapid and effective adjunctive methods in determining the extent of bleeding and the site of injury, differentially diagnosing arterial or venous bleeding, and confirming the presence of active bleeding. Prabhakar et al^[16]^ concluded that ultrasound and CT are necessary auxiliary equipment in the diagnosis of acute vascular injury. In contrast, angiography is the gold standard for the diagnosis of vascular injury^[17]^ and can accurately and specifically reflect vascular alignment and the site of injury. Hui et al^[8]^ concluded that angiography should be used to clarify the diagnosis when the patient is hemodynamically unstable and requires endovascular treatment at the same time. In addition, Shevlin et al^[18]^ recommended that the use of the Shevlin test to observe vascular injury.

When vascular injury is detected, the incision should be closed immediately with gauze packing, and depending on the condition, appropriate ancillary tests or even a dissection should be performed to clarify the diagnosis. At the same time, blood and fluid transfusions should be actively administered to maintain hemodynamic stability. Goodkin and Laska^[11]^ concluded that the idea of attempting to stop bleeding by compression is dangerous, delays treatment, and increases mortality. The expertise of a vascular surgeon is needed at this point and will significantly increase the success rate of vascular injury management.^[19]^

Commonly used surgical techniques include vascular repair, vascular anastomosis, vascular grafting, endovascular embolization, and stenting.^[20]^ A simple tear of a vessel can be repaired by lateral suturing. If stenosis occurs after repair, a vascular graft can be applied with the saphenous vein or an artificial vessel.^[20,21]^ Severe injuries of the common iliac artery that cannot be treated by vascular grafting can be treated by end-to-end anastomosis using the contralateral internal iliac artery. In contrast, ligation may be considered for severe injuries of the internal iliac artery. With the development of endovascular techniques, selective vascular embolization and interventions have become the treatment of choice for vascular injuries with advantages such as being minimally invasive and rapidly effective.^[9,10]^ Nam et al^[12]^ reported the successful treatment of a right common iliac artery tear complicating intervertebral disc surgery by applying endovascular balloon dilatation embolization followed by insertion of a stent to close the fissure. Gallerani et al^[10]^ reported several cases of vascular injury due to a history of previous disc surgery. To avoid this, preoperative familiarity with the depth of intervertebral pace and the disc quality should be grasped, and intraoperative manipulation should be as gentle as possible, without the need to pursue complete removal of the patient’s disc.^[11]^ According to Harbison^[1]^ that the key to treatment is to act quickly. This involves controlling bleeding and shock, followed by restoring hemodynamic stability as soon as possible. In our case, right after the occurrence of vascular injury, we immediately took effective measures to maintain hemodynamic stability by implementing blood and fluid transfusion, and actively contacting vascular surgery for adjunctive treatment after postoperative shock symptoms appeared. Due to this, angiography was expeditiously performed to discover the site of vascular injury, and endovascular treatment was initiated with stent placement, resulting in a successful intervention without serious consequences, with the patient discharged within a short time after symptomatic treatment.

Although the incidence of vascular injury during lumbar fusion surgery is low, it is often easily overlooked during surgery. Physicians should always be alert to the risk of vascular injury and master its clinical characteristics. Once injury is suspected, active and effective measures should immediately be taken for diagnosis and treatment to avoid serious adverse consequences.

## Acknowledgments

The authors gratefully acknowledge the patient who agreed to participate in this study, as well as Mr. Yunxiang Hu for his assistance during the manuscript preparation.

## Author contributions

HW contributed to study conception and design. JHW collected, analyzed clinical data and wrote the manuscript. YXH were involved in submitting and revising the paper. The final version of manuscript was read and approved by all authors.

**Conceptualization:** Jianhe Wang, Hong Wang.

**Data curation:** Jianhe Wang, Hong Wang.

**Formal analysis:** Jianhe Wang, Hong Wang.

**Funding acquisition:** Jianhe Wang, Hong Wang.

**Investigation:** Jianhe Wang, Hong Wang.

**Methodology:** Jianhe Wang, Hong Wang.

**Project administration:** Jianhe Wang, Hong Wang.

**Resources:** Jianhe Wang, Hong Wang.

**Software:** Jianhe Wang, Hong Wang.

**Supervision:** Jianhe Wang, Hong Wang.

**Validation:** Jianhe Wang, Hong Wang.

**Visualization:** Jianhe Wang, Hong Wang.

**Writing – original draft:** Jianhe Wang, Yunxiang Hu, Hong Wang.

**Writing – review & editing:** Jianhe Wang, Yunxiang Hu, Hong Wang.

## References

[1] Harbison SP. Major vascular complications of intervertebral disc surgery. Ann Surg. 1954;140:342–8. 13198071 10.1097/00000658-195409000-00010 PMC1609760

[2] Lacombe M. [Vascular complications of lumbar disk surgery]. Ann Chir. 2006;131:583–9. 16872577 10.1016/j.anchir.2006.06.010

[3] Brau SA Delamarter RB Schiffman ML. Vascular injury during anterior lumbar surgery. Spine J. 2004;4:409–12. 15246301 10.1016/j.spinee.2003.12.003

[4] Baker JK Reardon PR Reardon MJ. Vascular injury in anterior lumbar surgery. Spine. 1993;18:2227–30. 8278837 10.1097/00007632-199311000-00014

[5] Postacchini R Cinotti G Postacchini F. Injury to major abdominal vessels during posterior lumbar interbody fusion. A case report and review of the literature. Spine J. 2013;13:e7–11. 10.1016/j.spinee.2012.11.016 23219458

[6] Spittell J A JR Palumbo PJ Love JG. Arteriovenous fistula complicating lumbar-disk surgery. N Engl J Med. 1963;268:1162–5. 13990200 10.1056/NEJM196305232682104

[7] Fruhwirth J Koch G Amann W. Vascular complications of lumbar disc surgery. Acta Neurochir (Wien). 1996;138:912–6. 8890986 10.1007/BF01411278

[8] Hui YL Chung PC Lau WM. Vascular injury during a lumbar laminectomy. Chang Gung Med J. 2003;26:189–92. 12790223

[9] Erkut B Unlu Y Kaygin MA. Iatrogenic vascular injury during to lumbar disc surgery. Acta Neurochir (Wien). 2007;149:511–5; discussion 6. 17387429 10.1007/s00701-007-1132-2

[10] Gallerani M Maida G Boari B. High output heart failure due to an iatrogenic arterio- venous fistula after lumbar disc surgery. Acta Neurochir (Wien). 2007;149:1243–7; discussion 1247; discussion 7. 17987256 10.1007/s00701-007-1397-5

[11] Goodkin R Laska LL. Vascular and visceral injuries associated with lumbar disc surgery: medicolegal implications. Surg Neurol. 1998;49:358–70; discussion 370; discussion 70–2. 9537654 10.1016/s0090-3019(97)00372-8

[12] Nam TK Park SW Shim HJ. Endovascular treatment for common iliac artery injury complicating lumbar disc surgery: limited usefulness of temporary balloon occlusion. J Korean Neurosurg Soc. 2009;46:261–4. 19844629 10.3340/jkns.2009.46.3.261 PMC2764027

[13] Bingol H Cingoz F Yilmaz AT. Vascular complications related to lumbar disc surgery. J Neurosurg. 2004;100(3 Suppl Spine):249–53. 15029913 10.3171/spi.2004.100.3.0249

[14] Desaussure RL. Vascular injury coincident to disc surgery. J Neurosurg. 1959;16:222–8. 13642113 10.3171/jns.1959.16.2.0222

[15] Mack JR. Major vascular injuries incident to intervertebral disk surgery. Am Surg. 1956;22:752–63. 13354945

[16] Prabhakar H Bithal PK Dash M. Rupture of aorta and inferior vena cava during lumbar disc surgery. Acta Neurochir (Wien). 2005;147:327–9; discussion 329; discussion 9. 15625589 10.1007/s00701-004-0405-2

[17] Kiguchi M O’rourke HJ Dasyam A. Endovascular repair of 2 iliac pseudoaneurysms and arteriovenous fistula following spine surgery. Vasc Endovascular Surg. 2010;44:126–30. 20034939 10.1177/1538574409352809

[18] Shevlin WA Luessenhop AJ Fox JL. Perforation of the anterior annulus during lumbar discectomy. Case report. J Neurosurg. 1973;38:514–5. 4696202 10.3171/jns.1973.38.4.0514

[19] Jeon SH Lee SH Choi WC. Iliac artery perforation following lumbar discectomy with microsurgical carbon dioxide laser: a report of a rare case and discussion on the treatment. Spine. 2007;32:E124–5. 17268256 10.1097/01.brs.0000254078.88358.33

[20] Skippage P Raja J Mcfarland R. Endovascular repair of iliac artery injury complicating lumbar disc surgery. Eur Spine J. 2008;17(Suppl 2):S228–31. 17712578 10.1007/s00586-007-0470-3 PMC2525903

[21] Papadoulas S Konstantinou D Kourea HP. Vascular injury complicating lumbar disc surgery. A systematic review. Eur J Vasc Endovasc Surg. 2002;24:189–95. 12217278 10.1053/ejvs.2002.1682

